# Sputum ACE2, TMPRSS2 and FURIN gene expression in severe neutrophilic asthma

**DOI:** 10.1186/s12931-020-01605-8

**Published:** 2021-01-07

**Authors:** Nazanin Zounemat Kermani, Woo-Jung Song, Yusef Badi, Ali Versi, Yike Guo, Kai Sun, Pank Bhavsar, Peter Howarth, Sven-Erik Dahlen, Peter J. Sterk, Ratko Djukanovic, Ian M. Adcock, Kian Fan Chung, U-BIOPRED Consortium, Uruj Hoda, Christos Rossios, Elisabeth Bel, Navin Rao, David Myles, Chris Compton, Marleen Van Geest, Peter Howarth, Graham Roberts, Diane Lefaudeux, Bertrand De Meulder, Aruna T. Bansal, Richard Knowles, Damijn Erzen, Scott Wagers, Norbert Krug, Tim Higenbottam, John Matthews, Veit Erpenbeek, Leon Carayannopoulos, Amanda Roberts, David Supple, Pim deBoer, Massimo Caruso, Pascal Chanez, Sven-Erik Dahlen, Ildikó Horváth, Nobert Krug, Jacek Musial, Thomas Sandström

**Affiliations:** 1 Data Science Institute, Imperial College London, London, UK; 2 National Heart and Lung Institute, Imperial College London, Dovehouse St, London, SW3 6LY UK; 3 Department of Allergy and Clinical Immunology, Asan Medical Center, University of Ulsan College of Medicine, Seoul, Korea; 4 Faculty of Medicine, Southampton University, Southampton, UK; 5 NIHR Southampton Respiratory Biomedical Research Unit, University Hospital Southampton, Southampton, UK; 6 Centre for Allergy Research, Karolinska Institute, Stockholm, Sweden; 7 Amsterdam University Medical Centers, University of Amsterdam, Amsterdam, Netherlands

**Keywords:** ACE2, FURIN, TMPRSS2, Severe asthma, Neutrophil

## Abstract

**Background:**

Patients with severe asthma may have a greater risk of dying from COVID-19 disease. Angiotensin converting enzyme-2 (ACE2) and the enzyme proteases, transmembrane protease serine 2 (TMPRSS2) and FURIN, are needed for viral attachment and invasion into host cells.

**Methods:**

We examined microarray mRNA expression of ACE2, TMPRSS2 and FURIN in sputum, bronchial brushing and bronchial biopsies of the European U-BIOPRED cohort. Clinical parameters and molecular phenotypes, including asthma severity, sputum inflammatory cells, lung functions, oral corticosteroid (OCS) use, and transcriptomic-associated clusters, were examined in relation to gene expression levels.

**Results:**

ACE2 levels were significantly increased in sputum of severe asthma compared to mild-moderate asthma. In multivariate analyses, sputum ACE2 levels were positively associated with OCS use and male gender. Sputum FURIN levels were significantly related to neutrophils (%) and the presence of severe asthma. In bronchial brushing samples, TMPRSS2 levels were positively associated with male gender and body mass index, whereas FURIN levels with male gender and blood neutrophils. In bronchial biopsies, TMPRSS2 levels were positively related to blood neutrophils. The neutrophilic molecular phenotype characterised by high inflammasome activation expressed significantly higher FURIN levels in sputum than the eosinophilic Type 2-high or the pauci-granulocytic oxidative phosphorylation phenotypes.

**Conclusion:**

Levels of ACE2 and FURIN may differ by clinical or molecular phenotypes of asthma. Sputum FURIN expression levels were strongly associated with neutrophilic inflammation and with inflammasome activation. This might indicate the potential for a greater morbidity and mortality outcome from SARS-CoV-2 infection in neutrophilic severe asthma.

## Background

Coronavirus disease 2019 (COVID-19) is posing an unprecedented impact on global health. COVID-19 infection caused by the SARS-CoV-2 virus has been reported to be more common or severe in those male subjects aged above 70 years old or those with pre-existing conditions such as obesity, hypertension and diabetes or those who are cigarette smokers [1–5]. A few studies have not reported a significant association between asthma and critical outcomes in COVID-19 patients [1–5]. However, a large population-based study of ~ 11,000 COVID-related deaths in the UK reported a higher risk of death in patients with severe asthma who developed COVID-19 disease but not in those with non-severe asthma [6], while another study of COVID-19 hospitalisations in the UK showed that adults with asthma, particularly those with non-allergic asthma, were at a higher risk of severe COVID-19 infection [7].

One of the potential determinants of the severity of SARS-CoV2 infection is the degree of activation and multiplication of this new emergent virus in human cells [8, 9]. The spike protein of SARS-CoV-2 is used by the virus to engage and bind its target cell receptor, the angiotensin converting enzyme 2 (ACE2) [10]. In addition, the spike protein needs to be activated and degraded by host cell enzyme proteases, such as transmembrane protease, serine 2 (TMPRSS2) and FURIN also known as PACE (Paired basic Amino acid Cleaving Enzyme) [11, 12], which are essential for the proteolytic activation of SARS-CoV-2 in human airway epithelial cells [13]. Because this is the mechanism by which SARS-CoV-2 infects and actively replicates in respiratory tract cells, we tested the hypothesis that the expression of these receptors and proteases needed by the virus may vary according to asthma severity or to molecular phenotypes.

Our approach was to study the participants in the U-BIOPRED (**U**nbiased **BIO**markers **P**redictive of **RE**spiratory **D**isease outcomes) study [14], which was a cohort study for analysis of clinical and molecular features of severe asthma. Therefore, these patients had undergone detailed clinical and physiologic characterisation, with gene profiling studies of various lung and blood samples. We examined the transcriptomic data obtained from three different compartments of airway cells, namely, bronchial brushings, bronchial biopsies and sputum-derived cells from patients with severe asthma compared to those with mild-to-moderate asthma and to non-asthmatic controls and analysed the expression of genes that encode for ACE2, TMPRSS2 and FURIN. We also examined the relationship of the gene expression with demographic and clinical parameters, and with asthma molecular phenotypes or transcriptomic-associated clusters (TACs), previously identified in the U-BIOPRED [15].

## Methods

### Subjects

The U-BIOPRED cohort consisted of 3 groups: (i) severe asthma (SA) including current and ex-smokers, (ii) mild/moderate non-smoking asthmatics (MMA) and (iii) non-smoking healthy volunteers (HV). Clinical data, such as age, sex, body mass index (BMI), smoking history, and oral corticosteroid (OCS) use and comorbidities were collected, as previously described [14]. Demographic data of the 3 groups are shown in Additional file 1: Table S1. Expression profiling was performed using U133Plus 2.0 microarray (Affymetrix, Santa Clara, CA) on total RNA extracted from sputum cells, brushings of the lower airways, and bronchial biopsies. The transcriptomic data was assessed by multiarray average normalization. Atopy was defined by at least 1 inhalant allergen being positive on skin prick test or by high levels of serum allergen specific IgE. Participants gave signed informed consent to participate in the study, which was approved by the local Ethics Committees.

### Data analysis

Data were downloaded from the tranSMART platform [16]. We analysed the gene expression of ACE2, TMPRSS2, and FURIN in the lower airway samples obtained from asthmatics and healthy subjects. Gene expression levels and inflammatory cell counts in sputum were log-transformed to normalize their distribution. One-way ANOVA and Tukey’s multiple comparison tests were utilized to examine differences of the gene expression levels of ACE2, TMPRSS2 and FURIN across the 3 groups (SA, MMA and HV) or the 3 TACs [15]. Linear regression analyses were used to examine baseline demographic and clinical parameters in relation to each gene expression level. Multivariate linear regressions were conducted to identify baseline parameters significantly correlated with each gene expression level, with adjustment for age, gender and any parameters showing *p* values < 0.05 in univariate analyses; when both sputum and blood eosinophils (%) or neutrophils (%) showed *p* values < 0.05, sputum values were chosen for the multivariate model. The Benjamini–Hochberg procedure controlled for false discovery rate. Spearman's rank correlation was used for correlation analysis.

### Expression of signatures by gene set variation analysis

Gene set variation analysis (GSVA) was used to calculate sample-wise enrichment scores (ESs) [17] for six asthma-associated gene signatures. These gene sets each relate to a specific aspect of airway inflammation and asthma pathogenesis (Additional file 1: Table S2). The correlations between ESs and expression of ACE2, TMPRSS2, and FURIN were calculated using Spearman's correlation.

## Results

### Expression of ACE2

**Fig. 1:**
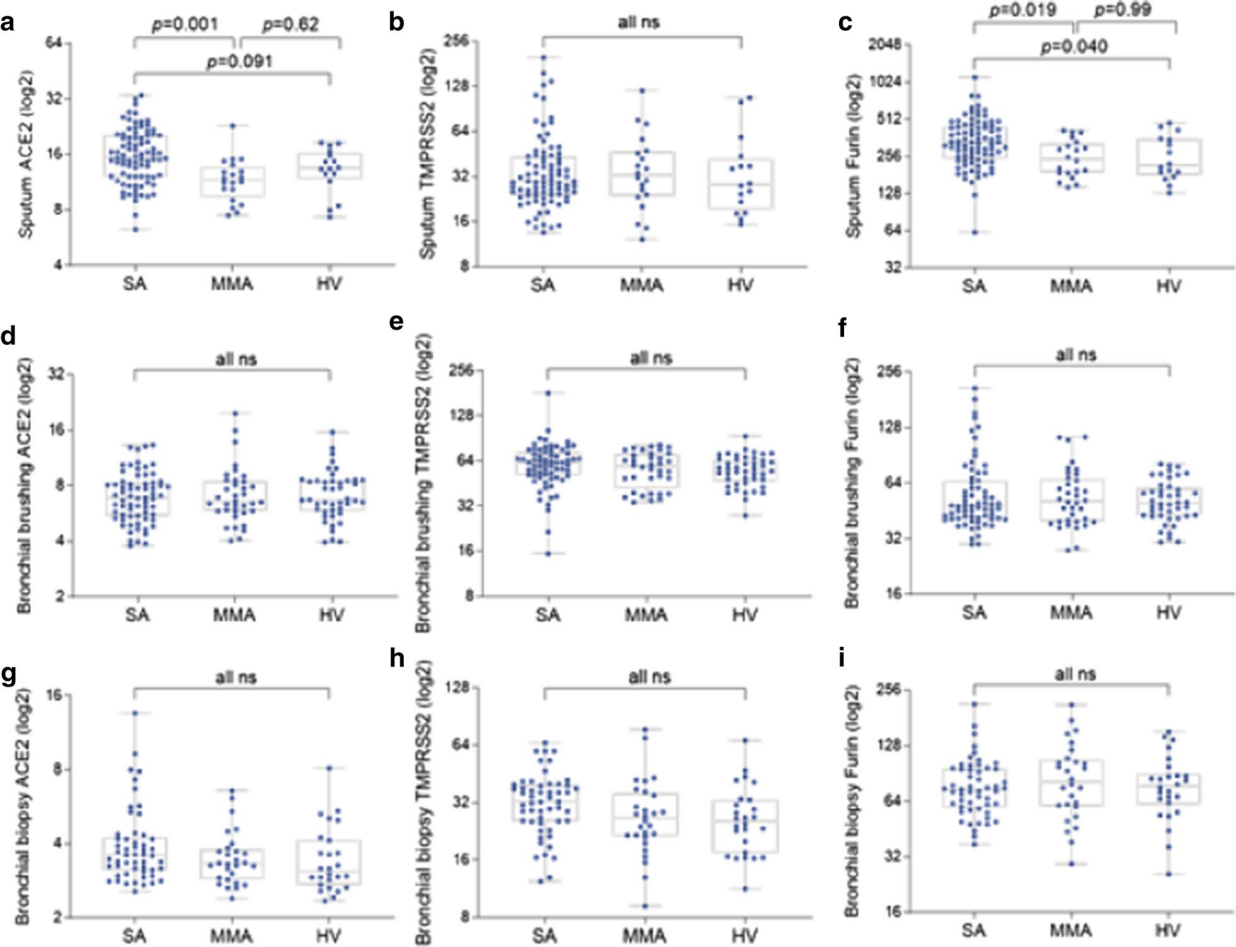
Comparison of ACE2, TMPRSS2 and FURIN gene expression levels (box-and-whisker plots showing median and interquartile range) between healthy volunteers and asthmatics in different airway compartments. ACE2 (**a**, **d**, **g**), TMPRSS2 (**b**, **e**, **h**) and FURIN (**c**, **f**, **i**) mRNA expression was assessed in sputum (**a**–**c**), bronchial brushings (**d**–**f**), and bronchial biopsies (**g**–**i**) compartments from healthy volunteers and asthmatics of different severity. *SA* severe asthmatics, *MMA* mild-to-moderate asthmatics, *HV* healthy volunteers, *ns* not significant (*p* > 0.05). *P* values were determined by one-way ANOVA and Tukey’s multiple comparison tests

**Fig. 2:**
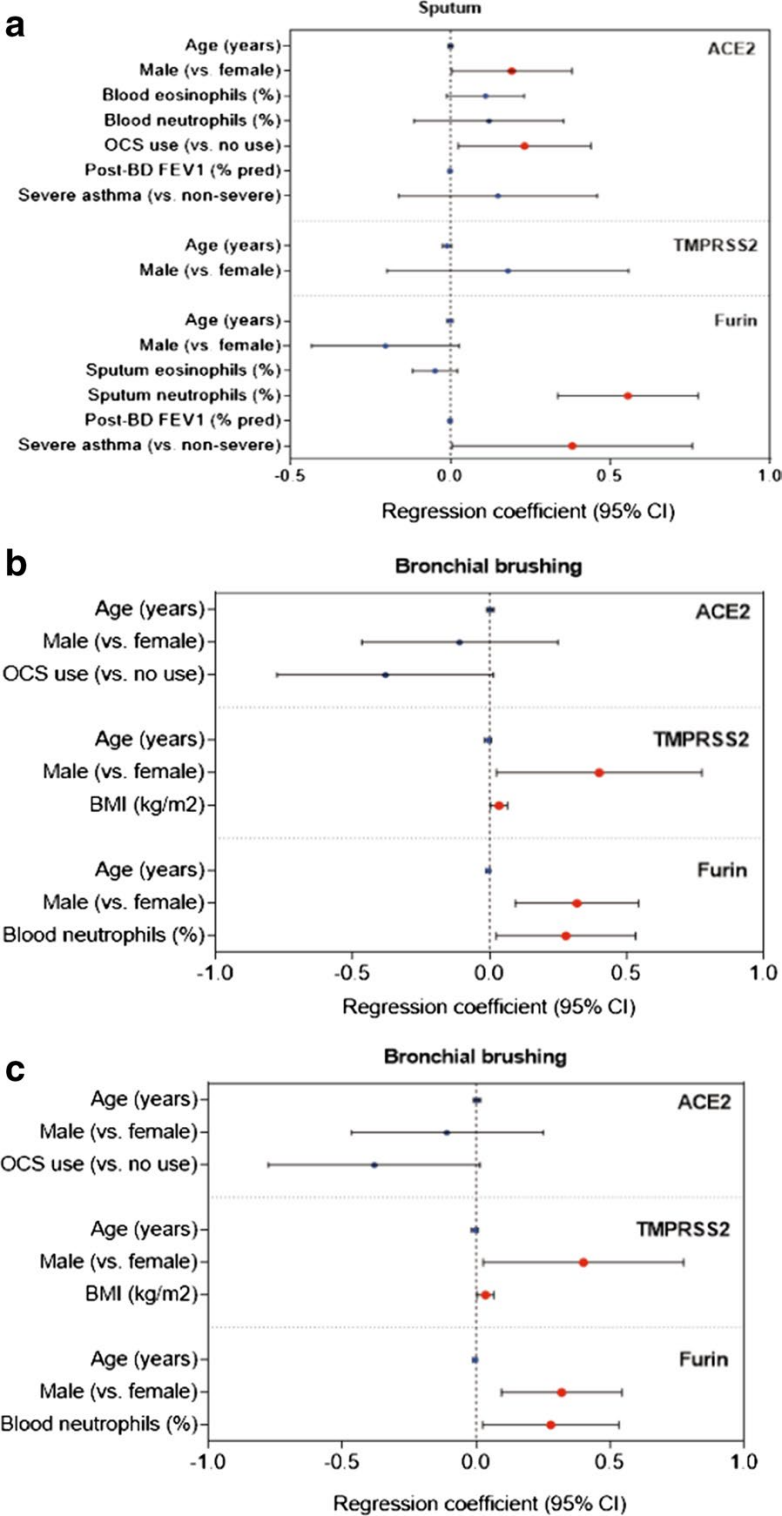
Forest plots showing associations between gene expression of ACE2, TMPRRS2 and FURIN, and baseline characteristics in **a** sputum, **b** bronchial brushings and **c** bronchial biopsies of asthmatics. Regression coefficients (95% confidence intervals) and *p* values were determined using multivariate linear regression tests for each gene expression level (as a dependent variable) and baseline parameters including age, gender, and those showing *p* values < 0.05 in univariate regression tests (shown in Table 1) as independent variables. All independent variables included in each multivariate model are presented. Gene expression levels and inflammatory cell counts (in sputum and blood) were log-transformed to normalize the distribution. Statically significant parameters (*p* < 0.05) are indicated with red color in symbol. *FEV*_*1*_ forced expiratory volume in the first second, *OCS* oral corticosteroid use, *post-BD* post bronchodilator

In sputum, ACE2 levels were significantly higher in SA than in MMA (*p* = 0.001) but was not different from HV (*p* = 0.091) (Fig. 1a). Among asthmatics, ACE2 levels were significantly associated with blood eosinophils (%), blood neutrophils (%), post-bronchodilator (post-BD) FEV_1_ (% predicted), OCS use, and severe asthma in univariate linear regression (Table 1). In multivariate regression analyses, ACE2 expression levels were positively associated with male gender (correlation coefficient: 0.192; 95% confidence interval [95% CI]: 0.004 to 0.381; *p* = 0.045) and OCS use (0.232; 95% CI: 0.025 to 0.440; *p* = 0.029) (Fig. 2a). The effects of severe asthma or post-BD FEV_1_ (% predicted) on ACE2 levels became non-significant when adjusted for OCS use (data not shown). Positive association between sputum ACE levels and OCS use is presented in a box and whisker plot (Additional file 1: Fig S1).

**Table 1:** SARS-CoV-2 entry-related gene expressions in the lower airways and their correlations with baseline parameters in asthmatics

	Sputum (n = 104)			Bronchial brushing (n = 103)			Bronchial biopsy (n = 81)		
Parameters	ACE2†	TMPRSS2†	FURIN†	ACE2†	TMPRSS2†	FURIN†	ACE2†	TMPRSS2†	FURIN†
Age (years)	0.009 (− 0.005 to 0.024)	− 0.012 (− 0.025 to 0.002)	0.009 (− 0.005 to 0.023)	− 0.006 (− 0.018 to 0.007)	-0.0005 (− 0.014 to 0.013)	0.002 (− 0.012 to 0.016)	0.003 (− 0.012 to 0.019)	0.012 (− 0.005 to 0.028)	0.088 (− 0.012 to 0.016)
Male (vs. female)	0.320 (− 0.078 to 0.718)	0.194 (− 0.186 to 0.575)	− 0.263 (− 0.637 to 0.112)	− 0.183 (− 0.537 to 0.170)	0.350 (− 0.018 to 0.719)	0.533 (0.143 to 0.924)**	0.086 (− 0.328 to 0.500)	0.281 (− 0.156 to 0.718)	0.183 (− 0.258 to 0.624)
Ever smoking (vs. never)	− 0.077 (− 0.485 to 0.332)	0.233 (− 0.153 to 0.618)	0.104 (− 0.279 to 0.487)	0.252 (− 0.129 to 0.632)	0.191 (− 0.212 to 0.594)	0.225 (− 0.210 to 0.659)	0.482 (0.031 to 0.933)*	0.218 (− 0.274 to 0.710)	0.225 (− 0.210 to 0.659)
BMI (kg/m^2^)	0.009 (− 0.029 to 0.048)	0.024 (− 0.012 to 0.060)	0.013 (− 0.023 to 0.049)	0.013 (− 0.016 to 0.043)	0.034 (0.003 to 0.064)*	− 0.014 (− 0.048 to 0.019)	− 0.035 (− 0.070 to 0.00001)	0.004 (− 0.035 to 0.042)	− 0.014 (− 0.048 to 0.019)
Sputum eosinophils (%)†	0.0001 (− 0.056 to 0.056)	0.031 (− 0.065 to 0.126)	− 0.080 (− 0.152 to − 0.008)*	− 0.080 (− 0.202 to 0.041), n = 24	− 0.102 (− 0.236 to 0.033), n = 24	0.033 (− 0.153 to 0.219), n = 24	− 0.053 (− 0.235 to 0.130), n = 27	− 0.009 (− 0.216 to 0.197), n = 27	− 0.030 (− 0.206 to 0.146), n = 27
Sputum neutrophils (%)†	0.103 (− 0.061 to 0.267)	0.001 (− 0.263 to 0.264)	0.613 (0.438 to 0.788)***	− 0.248 (− 0.674 to 0.179), n = 24	− 0.029 (− 0.467 to 0.408), n = 24	0.159 (− 0.388 to 0.706), n = 24	0.096 (− 0.592 to 0.784), n = 27	− 0.293 (− 1.098 to 0.512), n = 27	0.040 (− 0.629 to 0.709), n = 27
Blood eosinophils (%)†	0.166 (0.041 to 0.291)*	0.072 (− 0.134 to 0.278)	− 0.009 (− 0.176 to 0.157)	− 0.057 (− 0.183 to 0.068)	0.015 (− 0.113 to 0.143)	0.121 (− 0.032 to 0.274)	− 0.018 (− 0.159 to 0.124)	0.010 (− 0.172 to 0.192)	− 0.011 (− 0.184 to 0.163)
Blood neutrophils (%)†	0.300 (0.071 to 0.529)*	− 0.307 (− 0.678 to 0.063)	0.389 (0.095 to 0.682)*	0.176 (− 0.027 to 0.380)	0.090 (− 0.120 to 0.300)	0.266 (0.014 to 0.519)*	0.179 (− 0.059 to 0.418)	0.398 (0.099 to 0.697)*	0.140 (− 0.160 to 0.440)
FeNO (ppb)	0.001 (− 0.005 to 0.006)	0.001 (− 0.004 to 0.007)	− 0.003 (− 0.008 to 0.002)	0.004 (− 0.002 to 0.010)	0.004 (− 0.002 to 0.011)	0.0002 (− 0.007 to 0.007)	− 0.001 (− 0.008 to 0.007)	− 0.005 (− 0.013 to 0.003)	0.0002 (− 0.007 to 0.007)
Serum periostin (ng/ml)	0.005 (− 0.008 to 0.018)	− 0.004 (− 0.016 to 0.009)	0.004 (− 0.008 to 0.018)	− 0.007 (− 0.021 to 0.008)	− 0.007 (− 0.223 to 0.009)	0.009 (− 0.007 to 0.025)	0.003 (− 0.015 to 0.022)	− 0.010 (− 0.030 to 0.010)	0.009 (− 0.007 to 0.025)
Atopy (vs. no)	0.010 (− 0.471 to 0.491)	0.254 (− 0.202 to 0.711)	− 0.312 (− 0.761 to 0.137)	0.276 (− 0.146 to 0.699)	0.061 (− 0.390 to 0.512)	− 0.013 (− 0.501 to 0.475)	0.345 (− 0.132 to 0.821)	− 0.383 (− 0.890 to 0.123)	− 0.013 (− 0.501 to 0.475)
Post− BD FEV1 (% predicted)	− 0.011 (− 0.019 to − 0.003)**	0.007 (− 0.001 to 0.015)	− 0.011 (− 0.019 to − 0.004)**	0.006 (− 0.002 to 0.014)	− 0.002 (− 0.010 to 0.007)	− 0.009 (− 0.018 to 0.0004)	− 0.006 (− 0.016 to 0.005)	− 0.007 (− 0.018 to 0.004)	− 0.009 (− 0.018 to 0.0004)
Allergic rhinitis (vs. no)	− 0.029 (− 0.440 to 0.381)	0.069 (− 0.314 to 0.452)	− 0.239 (− 0.603 to 0.126)	− 0.007 (− 0.376 to 0.362)	− 0.245 (− 0.628 to 0.137)	− 0.287 (− 0.701 to 0.127)	− 0.040 (− 0.464 to 0.385)	− 0.218 (− 0.667 to 0.231)	− 0.287 (− 0.701 to 0.127)
Nasal polyps (vs. no)	0.341 (− 0.081 to 0.763)	0.054 (− 0.352 to 0.459)	0.228 (− 0.168 to 0.623)	− 0.293 (− 0.694 to 0.108)	0.041 (− 0.363 to 0.446)	0.022 (− 0.432 to 0.475)	0.505 (0.027 to 0.982)*	0.341 (− 0.167 to 0.849)	0.022 (− 0.432 to 0.475)
OCS use (vs. no)	0.650 (0.273 to 1.027)**	0.008 (− 0.370 to 0.387)	0.351 (− 0.017 to 0.718)	− 0.408 (− 0.769 to − 0.047)*	0.014 (− 0.377 to 0.404)	0.044 (− 0.377 to 0.465)	− 0.302 (− 0.735 to 0.130)	0.356 (− 0.104 to 0.816)	0.044 (− 0.377 to 0.465)
Severe asthma (vs. non− severe)	0.882 (0.408 to 1.356)***	− 0.008 (− 0.487 to 0.472)	0.665 (0.210 to 1.120)**	− 0.063 (− 0.435 to 0.310)	0.205 (− 0.186 to 0.596)	0.005 (− 0.377 to 0.471)	0.321 (− 0.107 to 0.749)	0.391 (− 0.064 to 0.845)	0.047 (− 0.377 to 0.471)

Values indicate regression coefficients (95% confidence intervals) and *P* values from univariate linear regression analyses. Statistically significant correlations are marked bold and also indicated as follows: *p < 0.05; **p < 0.01; ***p < 0.001; otherwise non-significant (p > 0.05). †Variable log-transformed. *BMI* body mass index, *FeNO* fractional exhaled nitric oxide, *post-BD* post-bronchodilator, *OCS* oral corticosteroids

In bronchial brushing and biopsy, ACE2 levels were not significantly associated with the presence of asthma, or asthma severity (Fig. 1d, g). In bronchial brushing, univariate regression analysis showed an inverse relationship between ACE2 levels and OCS use (Table 1), but the relationship was not significant on multivariate analysis (− 0.380; 95% CI: − 0.775 to 0.015; *p* = 0.059; Fig. 2b). In bronchial biopsy, ever-smoking and ever-history of nasal polyps were significantly associated with ACE levels on univariate regression (Table 1), but both became non-significant on multivariate analysis (Fig. 2c).

### Expression of TMPRSS2

TMPRSS2 levels did not significantly differ between SA, MMA, and HV in any of airway compartments analysed (Fig. 1b, e and h). Among asthmatics, no baseline parameters were associated with sputum TMPRSS2 levels (Table 1 and Fig. 2a). However, TMPRSS2 levels in bronchial brushing were significantly related to male gender and body mass index (BMI) on multivariate analysis (0.401; 95% CI: 0.027 to 0.775; *p* = 0.036; and 0.035; 95% CI: 0.005 to 0.066; *p* = 0.022, respectively; Fig. 2b). In bronchial biopsies, TMPRSS2 levels were positively associated with blood neutrophils (%) (0.350; 95% CI: 0.040 to 0.661; *p* = 0.027; Fig. 2c).

### Expression of FURIN

**Fig. 3:**
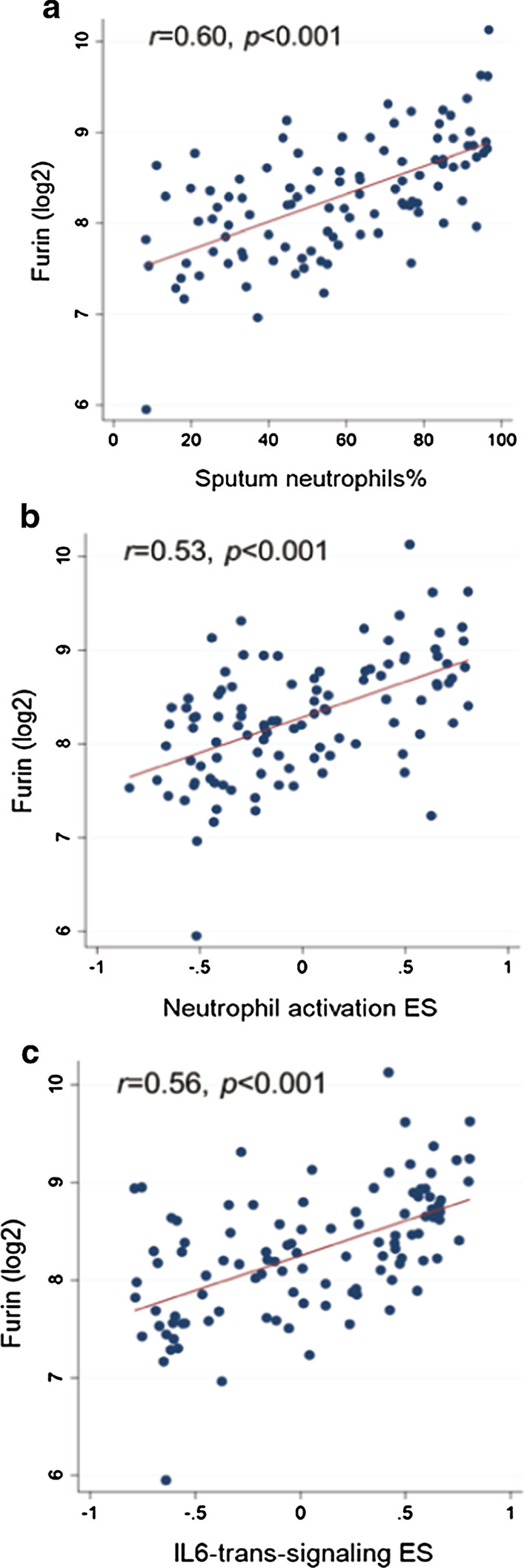
Scatter dot plots showing correlations between sputum FURIN gene expression levels and **a** neutrophils (%), **b** enrichment score (ES) of neutrophil activation gene signature and **c** ES of IL6-trans-signaling (IL6-TS) gene signature in sputum of asthmatics (n = 104). Correlation coefficients and *p* values were determined by Spearman’s tests

FURIN levels in sputum were significantly higher in SA than in MMA or HV (Fig. 1c). In univariate linear regression analyses, FURIN levels were significantly associated with sputum eosinophils (%), sputum neutrophils (%), blood neutrophils (%), post-BD FEV_1_ (% predicted), and severe asthma (Table 1). On multivariate analysis, sputum neutrophils (%) and severe asthma remained significantly associated with sputum FURIN levels (0.555; 95% CI: 0.336 to 0.775; *p* < 0.001; and 0.381; 0.005 to 0.757; *p* = 0.047, respectively) (Fig. 2a). The relationship between sputum FURIN levels and neutrophils (%) was not affected by further adjustments including OCS use (0.545; 95% CI: 0.324 to 0.767; *p* < 0.001). Spearman’s correlation coefficient (*r*) between sputum FURIN levels and neutrophils (%) was 0.60 (*p* < 0.001) (Fig. 3a).

In bronchial brushing, FURIN levels were positively associated with male gender and blood neutrophils (%) (0.320; 95% CI: 0.095 to 0.545; *p* = 0.006; and 0.279; 95% CI: 0.025 to 0.534; *p* = 0.032, respectively) (Fig. 2b). In bronchial biopsy, there was no association with any baseline parameters (Fig. 2c).

### Expression of ACE2, TMPRSS2 and FURIN in molecular phenotypes

**Fig. 4:**
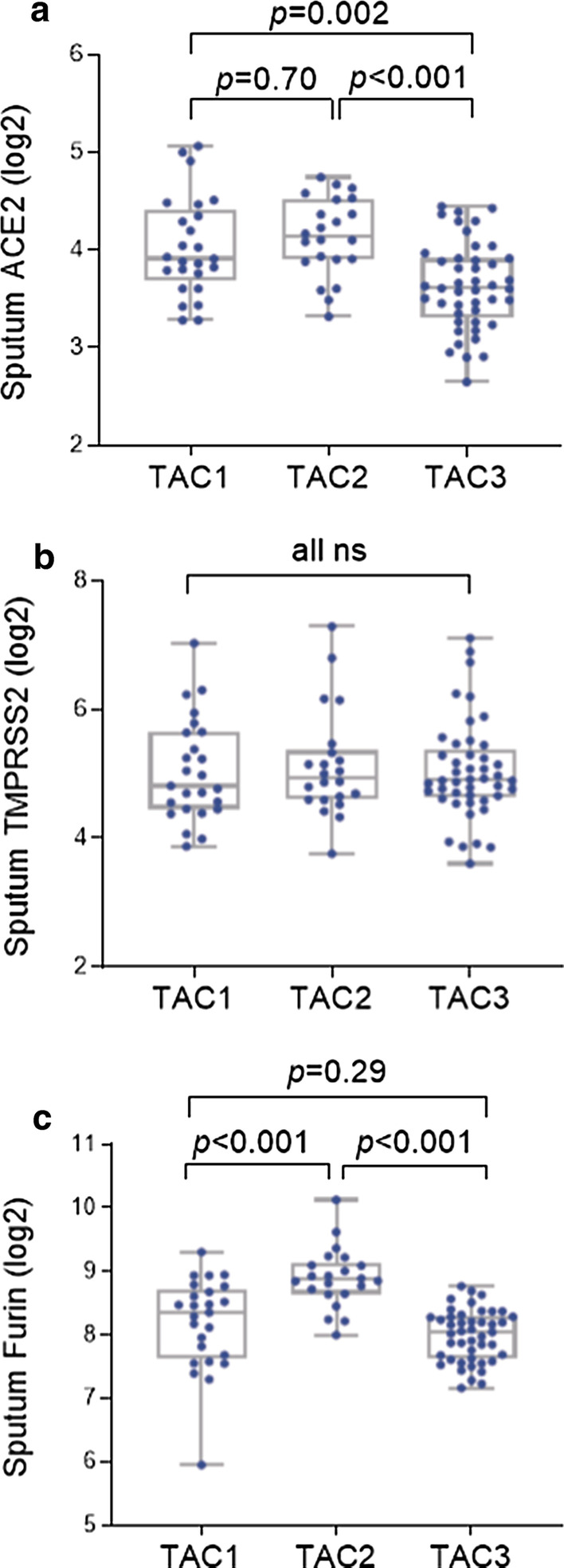
Comparison of **a** ACE2, **b** TMPRSS2 and **c** FURIN gene expression levels across transcriptomic-associated clusters (TACs) in sputum samples. Data shown as box-and-whisker plots showing median and interquartile range. *ns* not significant (*p* > 0.05). *P* values were determined by one-way ANOVA and Tukey’s multiple comparison tests

We compared the expression of these three genes in sputum amongst the three previously-identified TACs (Fig. 4) [15]. As previously reported, the analysis of sputum transcriptomics in the U-BIOPRED cohort produced three molecular endotypes of asthma [15]. ACE2 expression levels were significantly higher in TAC2 (an endotype characterized by sputum neutrophilic inflammation and inflammasome activation signature) and TAC1 (eosinophilic inflammation with an IL-13/T2-high pathway) than in TAC3 (pauci-granulocytic inflammation with oxidative phosphorylation pathway) (Fig. 4a). FURIN levels were significantly higher in TAC2 than in TAC1 and TAC3 (Fig. 4c). TMPRSS2 levels did not differ across TACs (Fig. 4b).

### Gene signatures and expression of ACE2, TMPRSS2 and FURIN

To further determine the relationship between gene signatures of interest and the expression levels of ACE2, TMPRSS2 and FURIN, we measured by GSVA the ESs of IL-13-Th2, eosinophil activation, IL-17, neutrophil activation, IL-6-trans-signalling (IL-6-TS), and inflammasome signatures (Additional file 1: Table S2). The ES of IL-13-Th2 signature was positively correlated with ACE2, TMPRSS2 and FURIN levels in the majority of compartments analysed (Table 2); however, the ES of eosinophil activation signature only weakly correlated with ACE2 in sputum (*r* = 0.18, *p* < 0.05) and with FURIN in bronchial biopsies (*r* = 0.26, *p* < 0.05). FURIN levels in all 3 compartments correlated with neutrophil activation and IL-6-TS signatures, with the highest correlation in sputum (Table 2). Spearman’s correlation coefficients (*r*) between sputum FURIN levels and the neutrophil activation and IL-6-TS signatures were *r* = 0.53 (*p* < 0.001) and *r* = 0.56 (*p* < 0.001), respectively (Fig. 3b and c). Inflammasome activation also correlated with FURIN levels in sputum (*r* = 0.49, *p* < 0.001) and in bronchial brushings (*r* = 0.30, *p* < 0.001; Table 2).

**Table 2:** Summary of correlations between SARS-CoV-2 entry-related gene expression and asthma-associated gene signatures

	Asthma-associated gene signature					
	IL13 Th2	Eosinophils	Th17	Neutrophil	IL-6-TS	Inflammasome
Sputum, n = 120						
ACE2	r = 0.38***	r = 0.18*	r = − 0.23*	*r* = 0.02	*r* = 0.14	*r* = 0.07
TMPRSS2	r = 0.57***	*r* = 0.15	*r* = − 0.06	*r* = 0.03	*r* = 0.02	*r* = 0.10
FURIN	*r* = 0.10	*r* = 0.13	*r* = − 0.16	r = 0.51***	r = 0.54***	r = 0.49***
Bronchial brushing, n = 149						
ACE2	*r* = 0.12	*r* = − 0.07	*r* = − 0.11	*r* = 0.0005	*r* = 0.11	r = 0.20*
TMPRSS2	r = 0.36***	*r* = − 0.04	*r* = − 0.10	*r* = − 0.06	*r* = − 0.07	*r* = − 0.06
FURIN	r = 0.30***	*r* = 0.14	*r* = 0.10	r = 0.24**	r = 0.30***	r = 0.30***
Bronchial biopsy, n = 108						
ACE2	r = 0.23*	*r* = 0.02	*r* = − 0.12	*r* = − 0.11	*r* = 0.01	*r* = 0.14
TMPRSS2	r = 0.37***	*r* = − 0.14	r = − 0.30**	*r* = − 0.14	*r* = − 0.01	*r* = − 0.01
FURIN	r = 0.33***	r = 0.26**	*r* = 0.06	r = 0.20*	r = 0.23*	*r* = 0.15

Association of ACE2, TMPRSS2, and FURIN expression with asthma-associated gene signatures were measured and tested using Spearman’s rank-order correlation. Statistically significant correlations are marked bold and also indicated as follows: ^*^p < 0.05; ^**^p < 0.01; ^***^p < 0.001. Otherwise non-significant (p > 0.05). IL-6-TS, IL-6-trans-signalling

## Discussion

By analysing the transcriptome of three lower airway compartments (bronchial biopsies, brushings, and sputum cells), we provide evidence for the over-expression of the factors that may determine the entry and activity of the SARS-CoV2 virus into host cells, particularly ACE2 and FURIN in sputum cells of severe asthmatics, compared to the mild-moderate asthmatics or healthy controls. This is in contrast to reports of no differences in the expression of ACE2, TMPRSS2 and FURIN between mild and severe asthma and healthy controls in sputum or in airway epithelial brushings and biopsies [18, 19]. Multivariate analysis showed that male gender and OCS use was associated with ACE2 expression in sputum, and male gender and BMI with TMPRSS2 and male gender with FURIN expression in bronchial brushings. Interestingly, male gender and obesity and also severe asthmatics who have had a recent course of OCS have been linked to a greater risk of death from SARS-CoV2 infection [6, 20]. Multivariate linear regression analysis also showed that the transcript levels of FURIN in sputum was significantly associated with severe asthma and sputum neutrophilia. Thus, patients with severe neutrophilic asthma, but not those with mild-moderate disease, may have the pathophysiological makeup that enhances the risk of severe infection by SARS-CoV-2. Our findings also indicate that the neutrophilic severe asthma may be more prone to a poorer outcome with SARS-CoV2. This finding is further supported by our associative analysis by molecular phenotype. Sputum ACE2 and FURIN transcripts were elevated in TAC2, an asthma endotype characterized by sputum neutrophilic inflammation and inflammasome activation signature.

The specific molecular cluster of TAC2 has been previously described in the U-BIOPRED cohort and is characterized by predominant sputum neutrophilic inflammation, inflammasome activation and neutrophilic activation [15]. In the present study, there was significantly higher expression of FURIN in sputum of TAC2, compared to TAC1 and TAC3. Furthermore, FURIN expression levels in sputum correlated highly with the expression of 3 gene signatures associated with neutrophil activation signature, inflammasome activation signature, and also a signature that reflected an IL-6-trans-signalling pathway that we have also previously reported [21]. The relationship between FURIN and neutrophils in sputum remained significant when adjusted for asthma severity, post-bronchodilator FEV_1_ (% predicted), or oral corticosteroid use. We also found modest but significant correlations between FURIN expression in bronchial brushings and bronchial biopsy and the expression of the neutrophil activation and IL-6 trans-signalling signature, and between FURIN expression in bronchial biopsy with the inflammasome signature. These findings lay emphasis on a potential link of FURIN with neutrophil activation, inflammasome, and IL-6 activation pathways, supported by high expression levels of IL-6 and neutrophil activation from lung epithelial cells infected with SARS-CoV2 in vitro and by serum levels of IL-6 being a strong predictor for respiratory failure in severe COVID-19 infection [22]. FURIN levels were not evaluated in the recent Severe Asthma Research Program-3 cohort analysis [19]. The study by Bradding et al. similarly found no significant difference of FURIN levels in airway epithelial brushing and biopsy by asthma presence or severity, but it did not examine sputum samples [18]. Interestingly, FURIN is most highly expressed in granulocytes such as neutrophils (https://www.proteinatlas.org/ENSG00000140564-FURIN/blood).

One could speculate that the presence of neutrophils in the airway mucosa could enhance the degradation of the spike protein on SARS-CoV-2 and facilitate viral entry into airway and inflammatory cells by FURIN [6]. In addition, neutrophils in the airways may be an important site for the propagation of SARS-CoV2 down the airways to the respiratory epithelium, that could lead to the development of pneumonia, a process that leads to hypoxaemia and more severe disease. Indeed, it has been recently shown that SARS-CoV2 can directly induce the release of neutrophil extracellular traps (NET) from neutrophils, which is in accord with the finding of NET concentration in the lungs of those who have died of COVID-19 infection was raised [23].

We found increased expression of ACE2 in sputum cells of the TAC1 eosinophilic phenotype that had an enrichment of gene signatures for IL-13/ Th2 inflammation and of the TAC2 phenotype, compared to TAC3, a pauci-granulocytic phenotype with increased metabolic and mitochondrial function genes [15]. ACE2 and TMPRSS2 expression in sputum were correlated with expression levels of the IL-13-Th2 signature. In bronchial biopsies, the levels of the 3 genes were all modestly correlated with that signature. On the other hand, atopy and sputum eosinophil counts were not significantly correlated with ACE2, TMPRSS2 and FURIN transcripts. We therefore have not confirmed the reports that ACE2 expression was reduced in mild-moderate asthmatics with T2-high compared to T2-low [24], and that ACE2 gene expression is positively correlated with Th2 gene expression in a group of asthmatics from mild to severe asthma [18].

There are significant differences between the asthma cohorts that have been published so far that have looked at the expression of ACE2, TMPRSS2 and FURIN as compared to healthy controls [18]. First, our U-BIOPRED cohort represents a large proportion of patients with very severe asthma as reflected by the large number of frequent exacerbations and the degree of airflow obstruction and the nearly 50% of patients on oral corticosteroid therapy [14], when compared to the other 3 cohorts that did not show any differences in expression of SARS-CoV2 entry genes [18, 24, 25]. In addition, our cohort had the oldest patients compared to these three studies, an important difference since old age is one of the most important predictor of death from COVID-19 infection [6]. There may also have been due to confounding by therapy (such as the use of OCS which was more highly prevalent in U-BIOPRED participants), the nature of the underlying T2 inflammatory process as well as the different but more comprehensive analytical approach taken in our study. Also, there may be differences in the asthma-driving mechanisms whereby the presence of differential contributions of Th2, Th17 and Th1 pathways in each asthmatic individual [26, 27], may determine the overall expression of the SARS-CoV-2 entry and of its activation genes.

Olfactory and taste disturbance is a common symptom of COVID-19, and the nasal epithelium is suggested to be a major route of viral infection [28, 29]. The gene expression levels of ACE2, TMPRSS2 and FURIN nasal brushings in our patients did not differ by asthma severity or molecular phenotype (data not presented), and also importantly, the levels were not significantly different in the presence of co-morbid nasal polyps (Additional file 1: Fig S2). However, the absolute gene expression levels of ACE2 and TMPRSS2 were higher in nasal brushings than in lower airway compartments (Additional file 1: Table S3), supporting a recent report that the nasal epithelium is a major site of infection [29]. Based on the expression patterns in the upper and lower airways, we suggest that severe asthmatics might have a higher risk of poorer outcomes from COVID-19, although the risk of infection via the nasal route is unlikely to be different by asthma severity.

Our study has major limitations. First, this is a cross-sectional study and could not determine causal relationships. Several features of severe asthma, such as sputum eosinophils and neutrophils, lung function or OCS use may be inter-related, but we used multivariate analyses to address this. Second, we did not find any positive correlations between smoking status (ever versus never-smokers) and target gene expressions, despite the fact that smoking can increase the expression of ACE2 in the airways [30–32]. However, it is likely that there were not enough current smokers in the severe asthma group (6.7%) to show any difference. Third, our analyses were only performed at gene expression levels, which should be confirmed at protein levels. On the other hand, the strength of our analysis is that it evaluated expression in a range of airway cell populations (epithelial brushing, bronchial biopsy and sputum cells), allowing an assessment of the molecular phenotypes. U-BIOPRED also had a large sample size of well-characterised severe asthma participants that was recruited at the same time as the healthy controls and mild-moderate asthmatics using the same pre-defined protocol. Finally, we have not examined protein expression of these SARS-CoV2 entry factors and particularly the protease activity of TMPRSS2 and FURIN.

In conclusion, we found higher gene expression levels of ACE2 and FURIN in sputum of severe asthma compared to those of non-severe asthma. Sputum FURIN levels highly correlated with sputum neutrophils and were higher in an asthma endotype characterized by sputum neutrophilia and inflammasome activation signature. Our data also supports the notion that the airway neutrophil may be a site of or potentiate the invasion by the SARS-CoV2 virus, thus increasing virus load and replication in patients with sputum neutrophilia. More importantly, the data also indicates that the severe neutrophilic asthma may be at risk of a poorer outcome if infected with SARS-CoV2 through the upregulation of FURIN, involved in the degradation of the SARS spike protein.

## Supplementary Information

**Additional file 1: Figure S1.** Comparison of sputum ACE2 gene expression levels (box-and-whisker plots showing median and interquartile range) by OCS use in asthmatics. P value was determined using unpaired t-test. **Fig S2.** Comparison of ACE2, TMPRSS2 and furin gene expression levels (box-and-whisker plots showing median and interquartile range) according to nasal polyps in nasal brushing. ns, not significant (p > 0.05). **Table S1**. Baseline characteristics of patients with asthma and healthy controls according to the site of mRNA collection. **Table S2**. Six asthma-associated gene sets analyzed in this study. **Table S3.** Absolute expression levels of ACE2, TMPRSS2 and Furin genes in different airway compartments

## Data Availability

The datasets generated and analysed during the current study are not publicly available due to being managed by U-BIOPRED consortium but are available from the corresponding author on reasonable request.

## Abbreviations

ACE2: Angiotensin converting enzyme-2
BD: Bronchodilator
ES: Expression score
FEV1: Forced expiratory volume in one second
GSVA: Gene set variation analysis
HV: Healthy volunteers
IL-6: Interleukin-6
MMA: Mild–moderate asthma
OCS: Oral corticosteroid
SA: Severe asthma
T-2: Type-2 inflammation
TAC: Transcriptomic-associated cluster
Th-2: T-helper 2 cell
Th-17: T-helper 17 cell
TMPRSS2: Transmembrane protease serine 2
U-BIOPRED: Unbiased BIOmarkers Predictive of REspiratory Disease

## References

[1] Guan WJ, Liang WH, Zhao Y, Liang HR, Chen ZS, Li YM (2020). Comorbidity and its impact on 1590 patients with COVID-19 in China: a nationwide analysis. Eur Respir J..

[2] Wu Z, McGoogan JM (2020). Characteristics of and important lessons from the coronavirus disease 2019 (COVID-19) outbreak in China: summary of a report of 72314 cases from the Chinese Center for Disease Control and Prevention. JAMA..

[3] Docherty AB, Harrison EM, Green CA, Hardwick HE, Pius R, Norman L (2020). Features of 20 133 UK patients in hospital with covid-19 using the ISARIC WHO Clinical Characterisation Protocol: prospective observational cohort study. BMJ..

[4] Petrilli CM, Jones SA, Yang J, Rajagopalan H, O’Donnell L, Chernyak Y (2020). Factors associated with hospital admission and critical illness among 5279 people with coronavirus disease 2019 in New York City: prospective cohort study. BMJ..

[5] Yang X, Yu Y, Xu J, Shu H, Xia J, Liu H (2020). Clinical course and outcomes of critically ill patients with SARS-CoV-2 pneumonia in Wuhan, China: a single-centered, retrospective, observational study. Lancet Respir Med.

[6] Williamson EJ, Walker AJ, Bhaskaran K, Bacon S, Bates C, Morton CE (2020). Factors associated with COVID-19-related death using OpenSAFELY. Nature..

[7] Zhu Z, Hasegawa K, Ma B, Fujiogi M, Camargo CA, Liang L (2020). Association of asthma and its genetic predisposition with the risk of severe COVID-19. J Allergy Clin Immunol..

[8] Fajnzylber J, Regan J, Coxen K, Corry H, Wong C, Rosenthal A (2020). SARS-CoV-2 viral load is associated with increased disease severity and mortality. Nat Commun.

[9] Pujadas E, Chaudhry F, McBride R, Richter F, Zhao S, Wajnberg A (2020). SARS-CoV-2 viral load predicts COVID-19 mortality. Lancet Respir Med.

[10] Yan R, Zhang Y, Li Y, Xia L, Guo Y, Zhou Q (2020). Structural basis for the recognition of SARS-CoV-2 by full-length human ACE2. Science.

[11] Hoffmann M, Kleine-Weber H, Schroeder S, Krüger N, Herrler T, Erichsen S (2020). SARS-CoV-2 cell entry depends on ACE2 and TMPRSS2 and is blocked by a clinically proven protease inhibitor. Cell.

[12] Coutard B, Valle C, de Lamballerie X, Canard B, Seidah NG, Decroly E (2020). The spike glycoprotein of the new coronavirus 2019-nCoV contains a furin-like cleavage site absent in CoV of the same clade. Antiviral Res.

[13] Bestle D, Heindl MR, Limburg H, Van Lam van T, Pilgram O, Moulton H (2020). TMPRSS2 and furin are both essential for proteolytic activation of SARS-CoV-2 in human airway cells. Life Sci Alliance..

[14] Shaw DE, Sousa AR, Fowler SJ, Fleming LJ, Roberts G, Corfield J (2015). Clinical and inflammatory characteristics of the European U-BIOPRED adult severe asthma cohort. Eur Respir J.

[15] Kuo C-HS, Pavlidis S, Loza M, Baribaud F, Rowe A, Pandis I (2017). T-helper cell type 2 (Th2) and non-Th2 molecular phenotypes of asthma using sputum transcriptomics in U-BIOPRED. Eur Respir J..

[16] Athey BD, Braxenthaler M, Haas M, Guo Y (2013). tranSMART: an open source and community-driven informatics and data sharing platform for clinical and translational research. AMIA Jt Summits Transl Sci Proc AMIA Summit Transl Sci.

[17] Hänzelmann S, Castelo R, Guinney J (2013). GSVA: gene set variation analysis for microarray and RNA-seq data. BMC Bioinf.

[18] Bradding P, Richardson M, Hinks TSC, Howarth PH, Choy DF, Arron JR (2020). ACE2, TMPRSS2 and furin gene expression in the airways of people with asthma—implications for COVID-19. J Allergy Clin Immunol..

[19] Peters MC, Sajuthi S, Deford P, Christenson S, Rios CL, Montgomery MT (2020). COVID-19-related genes in sputum cells in asthma. Relationship to demographic features and corticosteroids. Am J Respir Crit Care Med..

[20] Docherty AB, Harrison EM, Green CA, Hardwick HE, Pius R, Norman L (2020). Features of 20 133 UK patients in hospital with covid-19 using the ISARIC WHO clinical characterisation protocol: prospective observational cohort study. BMJ.

[21] Jevnikar Z, Ostling J, Ax E, Calven J, Thorn K, Israelsson E (2019). Epithelial IL-6 trans-signaling defines a new asthma phenotype with increased airway inflammation. J Allergy Clin Immunol..

[22] Blanco-Melo D, Nilsson-Payant BE, Liu WC, Uhl S, Hoagland D, Møller R (2020). Imbalanced host response to SARS-CoV-2 drives development of COVID-19. Cell.

[23] Herold T, Jurinovic V, Arnreich C, Lipworth BJ, Hellmuth JC, von Bergwelt-Baildon M, et al. Elevated levels of interleukin-6 and CRP predict the need for mechanical ventilation in COVID-19. J Allergy Clin Immunol. 2020;146(1):128–136.e4. 10.1016/j.jaci.2020.05.008 PMC7233239 32425269

[24] Veras FP, Pontelli MC, Silva CM, Toller-Kawahisa JE, de Lima M, Nascimento DC (2020). SARS-CoV-2-triggered neutrophil extracellular traps mediate COVID-19 pathology. J Exp Med..

[25] Kimura H, Francisco D, Conway M, Martinez FD, Vercelli D, Polverino F (2020). Type 2 inflammation modulates ACE2 and TMPRSS2 in airway epithelial cells. J Allergy Clin Immunol..

[26] Peters MC, Sajuthi S, Deford P, Christenson S, Rios CL, Montgomery MT (2020). COVID-19 related genes in sputum cells in asthma: relationship to demographic features and corticosteroids. Am J Respir Crit Care Med..

[27] Ziegler CGK, Allon SJ, Nyquist SK, Mbano IM, Miao VN, Tzouanas CN (2020). SARS-CoV-2 receptor ACE2 is an interferon-stimulated gene in human airway epithelial cells and is detected in specific cell subsets across tissues. Cell.

[28] Bhakta NR, Christenson SA, Nerella S, Solberg OD, Nguyen CP, Choy DF (2018). IFN-stimulated gene expression, Type 2 inflammation, and endoplasmic reticulum stress in asthma. Am J Respir Crit Care Med.

[29] Menni C, Valdes AM, Freidin MB, Sudre CH, Nguyen LH, Drew DA (2020). Real-time tracking of self-reported symptoms to predict potential COVID-19. Nat Med..

[30] Sungnak W, Huang N, Becavin C, Berg M, Queen R, Litvinukova M (2020). SARS-CoV-2 entry factors are highly expressed in nasal epithelial cells together with innate immune genes. Nat Med.

[31] Zhang H, Rostami MR, Leopold PL, Mezey JG, O'Beirne SL, Strulovici-Barel Y (2020). Expression of the SARS-CoV-2 ACE2 receptor in the human airway epithelium. Am J Respir Crit Care Med.

[32] Cai G, Bossé Y, Xiao F, Kheradmand F, Amos CI (2020). Tobacco smoking increases the lung gene expression of ACE2, the receptor of SARS-CoV-2. Am J Respir Crit Care Med.

[33] Smith JC, Sausville EL, Girish V, Yuan ML, Vasudevan A, John KM (2020). Cigarette smoke exposure and inflammatory signaling increase the expression of the SARS-CoV-2 receptor ACE2 in the respiratory tract. Dev Cell..

