# Correction to: Sputum ACE2, TMPRSS2 and FURIN gene expression in severe neutrophilic asthma

**DOI:** 10.1186/s12931-021-01642-x

**Published:** 2021-02-08

**Authors:** Nazanin Zounemat Kermani, Woo-Jung Song, Yusef Badi, Ali Versi, Yike Guo, Kai Sun, Pank Bhavsar, Peter Howarth, Sven-Erik Dahlen, Peter J. Sterk, Ratko Djukanovic, Ian M. Adcock, Kian Fan Chung, U-BIOPRED Consortium, Uruj Hoda, Christos Rossios, Elisabeth Bel, Navin Rao, David Myles, Chris Compton, Marleen Van Geest, Peter Howarth, Graham Roberts, Diane Lefaudeux, Bertrand De Meulder, Aruna T. Bansal, Richard Knowles, Damijn Erzen, Scott Wagers, Norbert Krug, Tim Higenbottam, John Matthews, Veit Erpenbeek, Leon Carayannopoulos, Amanda Roberts, David Supple, Pim deBoer, Massimo Caruso, Pascal Chanez, Sven-Erik Dahlen, Ildikó Horváth, Nobert Krug, Jacek Musial, Thomas Sandström

**Affiliations:** 1 Data Science Institute, Imperial College London, London, UK; 2 National Heart and Lung Institute, Imperial College London, Dovehouse St, London, SW3 6LY UK; 3 Department of Allergy and Clinical Immunology, Asan Medical Center, University of Ulsan College of Medicine, Seoul, Korea; 4 Faculty of Medicine, Southampton University, Southampton, UK; 5 NIHR Southampton Respiratory Biomedical Research Unit, University Hospital Southampton, Southampton, UK; 6 Centre for Allergy Research, Karolinska Institute, Stockholm, Sweden; 7 Amsterdam University Medical Centers, University of Amsterdam, Amsterdam, Netherlands

## Correction to: Respir Res (2021) 22:10. 10.1186/s12931-020-01605-8

Following publication of the original article [1], we were notified that Fig 2b and 2c were duplicated. Figure 2c should be the data from bronchial biopsy.

Also, in Additional Table S1, the values for Female Asthma and Control under Bronchial Brushing were swapped and the last table column should read “Bronchial biopsy” instead of “Bronchial Brushing”.

Originally published Fig. 2.
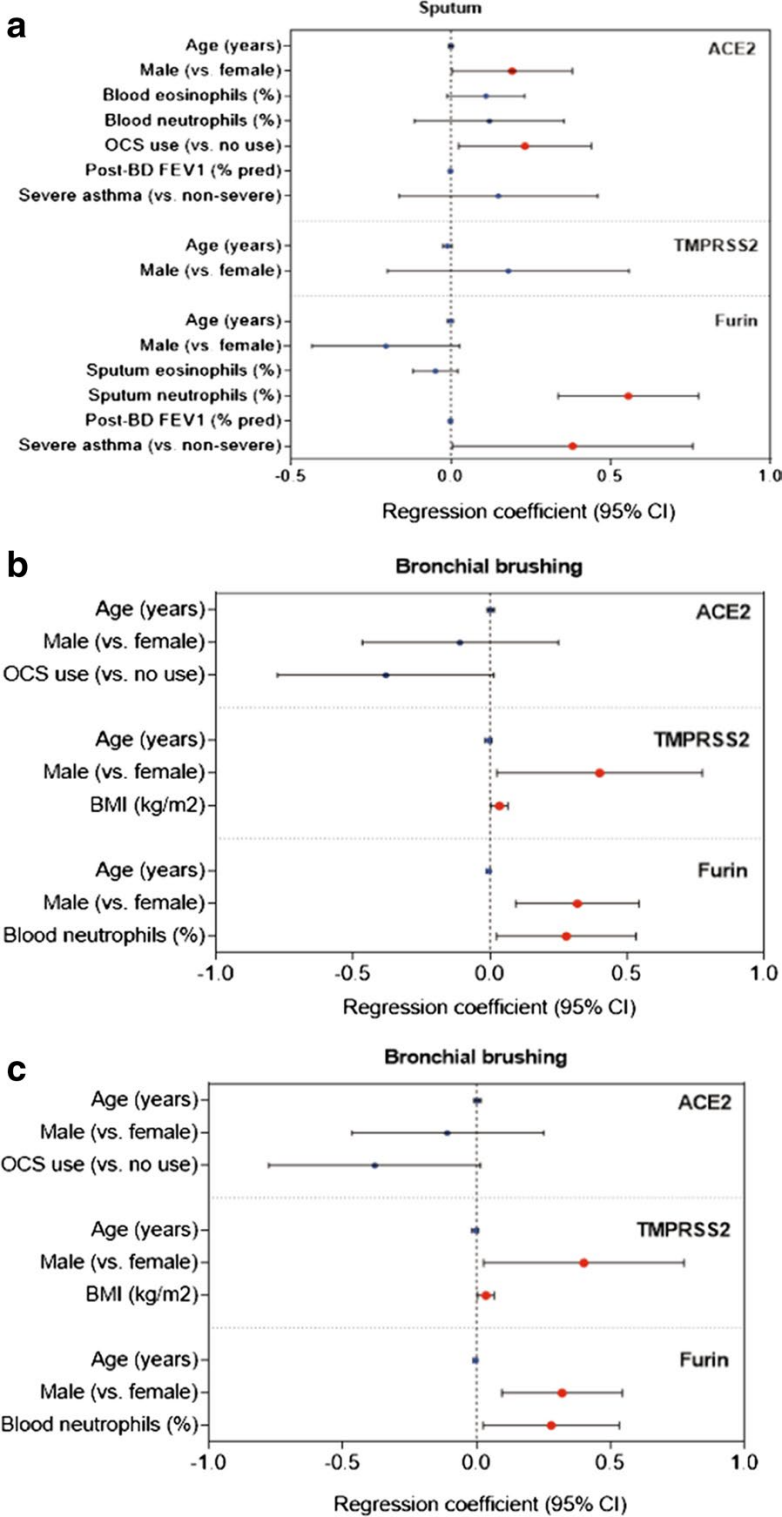


Corrected Fig. 2.
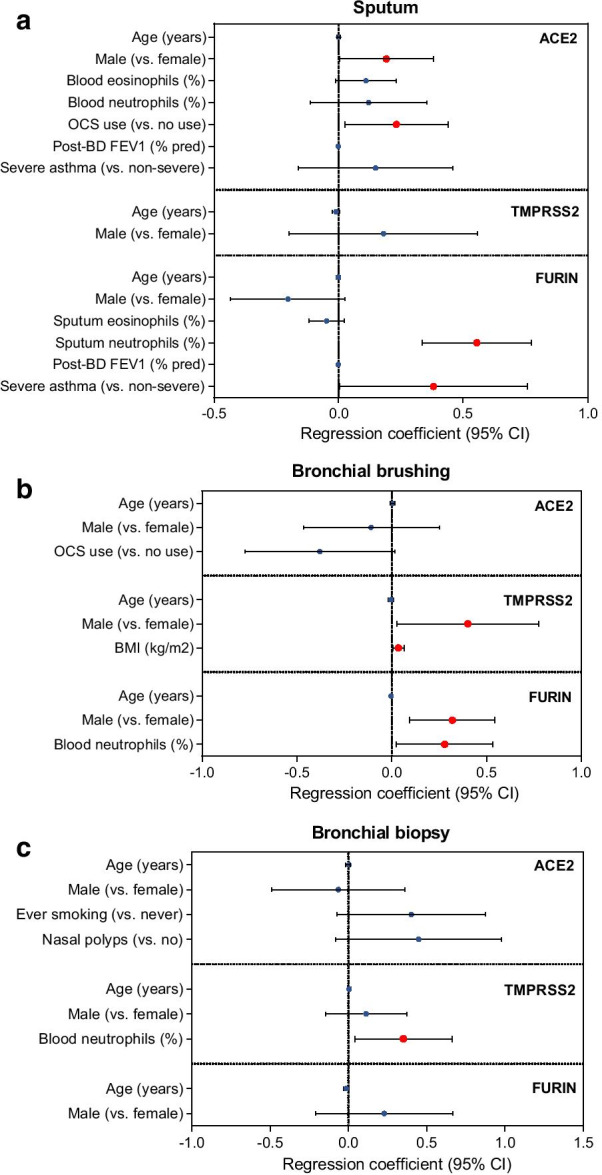


The original article has been corrected.
