# Supplementary material for: Sputum ACE2, TMPRSS2 and FURIN gene expression in severe neutrophilic asthma
Source: Respir Res. 2021 Jan 7;22:10. doi: 10.1186/s12931-020-01605-8 (PMC7788167; doi:10.1186/s12931-020-01605-8)
Supplement: Supplementary file 1 [file 12931_2020_1605_MOESM1_ESM.docx]

**Supplementary data files**

**Sputum ACE2, TMPRSS2 and FURIN gene expression in severe neutrophilic asthma** 

Nazanin Zounemat Kermani^1*^, Woo-Jung Song^2, 3*^, Yusef Badi^1,2^, Ali Versi^1,2^, Yike Guo^1^, Kai Sun^1^, Pank Bhavsar^2^, Peter Howarth^4^, Sven-Erik Dahlen^5^, Peter J Sterk^6^, Ratko Djukanovic^4^, Ian M Adcock^1,2^, Kian Fan Chung^1,2 ^on behalf the U-BIOPRED Consortium 

^1^Department of Computing & Data Science Institute, Imperial College London, United Kingdom; ^2^National Heart & Lung Institute, Imperial College London; ^3^Department of Allergy and Clinical Immunology, Asan Medical Center, University of Ulsan College of Medicine, Seoul, Korea; ^4^Faculty of Medicine, Southampton University, Southampton, UK and NIHR Southampton Respiratory Biomedical Research Unit, University Hospital Southampton, Southampton, UK;  ^5^Centre for Allergy Research, Karolinska Institute, Stockholm, Sweden; ^6^Amsterdam University Medical Centers, University of Amsterdam, Amsterdam, Netherlands.

*Contributed equally.

**Supplementary Figure S1-S2**

**Supplementary Tables S1-S3**

**U-BIOPRED Consortium members** 

**Supplementary Figure S1.** Comparison of sputum ACE2 gene expression levels (box-and-whisker plots showing median and interquartile range) by OCS use in asthmatics. P value was determined using unpaired t-test.

**Supplementary Fig S2.** Comparison of ACE2, TMPRSS2 and furin gene expression levels (box-and-whisker plots showing median and interquartile range) according to nasal polyps in nasal brushing. ns, not significant (p > 0.05).

 

**Supplementary Table S1. Baseline characteristics of patients with asthma and healthy controls according to the site of mRNA collection**

|  | Sputum |  |  | Bronchial brushing |  |  | Bronchial biopsy |  |  |
| --- | --- | --- | --- | --- | --- | --- | --- | --- | --- |
|  | Asthma | Control | *p* value | Asthma | Control | *p* value | Asthma | Control | *p* value |
| Subjects, n | 104 | 16 |  | 103 | 44 |  | 81 | 26 |  |
| Demographic parameters |  |  |  |  |  |  |  |  |  |
| Age (years) | 53 (45-61) | 39 (29-49) | 0.001 | 47 (34-58) | 34 (25.8-48.2) | 0.002 | 49 (36-58) | 37 (25-53.2) | 0.05 |
| Female | 58% | 25% | <0.001 | 52% | 36% | 0.093 | 54% | 38% | 0.03 |
| BMI (kg/m^2^) | 27.1 (24-31.8) | 25.7 (23.3-27.7) | 0.20 | 28.3 (24.7-33.1) | 24.6 (22.5-27) | <0.001 | 27.3 (24.4-32) | 24.4 (22.7-27.3) | 0.008 |
| Never smokers | 62% | 94% | <0.001 | 69% | 84% | 0.02 | 73% | 85% | 0.06 |
| Clinical parameters |  |  |  |  |  |  |  |  |  |
| Severe asthma | 81% | na |  | 65% | na |  | 65% | na |  |
| Asthma onset age (years) | 22 (6-40) | na |  | 15 (5-37) | na |  | 13 (3-38) | na |  |
| Atopy | 74% | 31% | <0.001 | 77% | 36% | <0.001 | 74% | 46% | 0.001 |
| Eczema | 32% | 0% | 0.007 | 43% | 7% | <0.001 | 42% | 8% | 0.001 |
| Allergic rhinitis | 53% | 13% | 0.003 | 62% | 16% | <0.001 | 60% | 19% | <0.001 |
| Nasal polyps | 33% | 7% | 0.037 | 27% | 7% | 0.007 | 24% | 4% | 0.024 |
| OCS use | 34% | 0% | <0.001 | 25% | 0% | <0001 | 24% | 0% | <0.001 |
| FEV1% predicted | 69.8 (53.9-85.7) | 104 (98.9-113.2) | <0.001 | 76.9 (63.9-95.4) | 100.7 (96.9-110.3) | <0.001 | 76.4 (62.2-92.8) | 99 (90.7-110.1) | <0.001 |
| FeNO (ppb) | 26 (16-46.5) | 17 (13.5-26.1) | 0.04 | 23.2 (16-54.5) | 16.2 (12.5-26.9) | 0.002 | 22.7 (15-45) | 24 (15-30.5) | 0.60 |
| Total IgE (IU/mL) | 103.5 (44.4-229.5) | 39.5 (14.5-99.4) | 0.02 | 105 (40.9-314.8) | 16 (7-61.7) | <0.001 | 111.5 (43-312.2) | 23 (7.5-92.8) | <0.001 |
| Serum periostin (ng/mL) | 49 (39.7-59.4) | 46.2 (43.9-51.5) | 0.70 | 45.9 (39.8-53.6) | 50.6 (45-59.5) | 0.1 | 44.2 (39.8-51.6) | 55.1 (45.2-60.8) | 0.01 |
| Blood eosinophils (x 10^9^/L) | 0.2 (0.1-0.4) | 0.1 (0.1-0.2) | <0.001 | 0.2 (0.1-0.3) | 0.1 (0.1-0.2) | <0.001 | 0.2 (0.1-0.3) | 0.1 (0.1-0.1) | 0.001 |
| Blood neutrophils (x 10^9^/L) | 4.4 (3.5-6.5) | 3.2 (2.8-5.1) | 0.04 | 4.2 (3-5.9) | 2.7 (2.2-3.6) | <0.001 | 4.3 (3.2-5.8) | 3 (2.1-3.8) | 0.001 |
| Sputum eosinophils (%) | 2.4 (0.2-12.5) | 0 (0-0.2) | <0.001 | 0.9 (0.2-6.5) | 0 (0-0.2) | <0.001 | 1.2 (0.3-5.7) | 0.2 (0-0.6) | 0.03 |
| Sputum neutrophils (%) | 58.1 (34.8-78.7) | 40.5 (19.6-68.9) | 0.05 | 52.3 (34.2-70.4) | 22.9 (15-50.9) | 0.004 | 52.3 (36.1-66.6) | 32.7 (14.2-44.2) | 0.03 |

BMI: body mass index; FEV1: forced expiratory volume in 1 second; FeNO: exhaled nitric oxide fraction; CRP: C-reactive protein. 

Variables are described in %, or median (interquartile range 25%-75%). The Chi-squared test was used for computing the *P* value of categorical variables. Normality of numerical variables was examined by the Shapiro-Wilk test. Mann-Whitney U test was used for non-normally distributed numerical variables and pairwise Student’s t-test for normally distributed numerical variables.

**Supplementary Table S2. Six asthma-associated gene sets analyzed in this study**

| Name of gene set | Gene | Reference |
| --- | --- | --- |
| IL13-Th2 | CST1, CCL26, PRB2, PRB1, PRB3, POSTN, PRB4, ITLN1, ALOX15, SH2D1B, CA2, NOS2, FCGBP, FOXA3, SPDEF, CAPN14, DUOXA2, CLDN5, PADI3, TSPAN8, ALPL, KCNJ16, FETUB, B3GNT6, CDH26, LRRC31, MUC13, VSIG2, CSTA, FAM3B, SLC9B2, NTRK1, KLF4, HPDL, SOCS1, TRNP1, HS3ST1, VWF, DUOX2, CISH, ATP13A5, ZNF808, RNASE4, CCBL1, SDCBP2, TMPRSS2, HYAL1, CCDC109B, FAM83D, TRAK1, TPK1, SLC7A1, CYP2C18, CDC42EP5, KCNS3, ADRA2A, MRAP2, SLC2A10, PPARG, FAM26E, ADCY4, WNT3, SLCO4A1, ALDH1A2, C10orf99, WDFY2 | (1) |
| Eosinophils activation | CLC, SIGLEC8, EMR1, EMR4P, LGAL312, HRH4, CEBPE, DACH1, VSTM1 | (2) |
| Th17 | KLRB1, RORC, PLXND1, CTSH, ALOX5, PTPN13, IL4I1, C11orf75, NEFL, HLF, JAKMIP2, DSE, LIMS1, HLA-DRB1, LTK, HLA-DRB4, USP10, NR1D1, LCAT, SAMD3, HSPG2 | (3) |
| Neutrophil activation | ABTB1, AMPD2, C5orf6, CCR3, CDA, CKLFSF2, CLC, CREB5, CTBS, DcR1, EST, FCGR2B, FCGR3B, FLJ10298, FPRL1, FRAT2, GPR27, GPR43, HSPA6, IL8RA, IL8RB, KIAA0779, KIAA1126, KRT23, LENG4, LENG5, MAD, MGC10500, MGC14126, MGC16353, MPPE1, MSCP, NCF4, NRBF-2, PHC2, PROK2, RALB, RNF141, SEC14L1, SEPX1, STX3A, TM4-B, VMP1, VNN2,XPO6 | (4) |
| IL-6-trans-signalling | TNFAIP6 PDE4B IL1R2 S100A9 S100A8 S100A12 CHI3L1 SPP1 | (5) |
| Inflammasome activation | IL1B, NLRP3, CASP1, CASP4, CASP5 | (6) |

**References**

1.	Alevy YG, Patel AC, Romero AG, Patel DA, Tucker J, Roswit WT, et al. IL-13–induced airway mucus production is attenuated by MAPK13 inhibition. The Journal of clinical investigation. 2012;122(12):4555-68.

2.	Leaker B, Malkov V, Mogg R, Ruddy M, Nicholson G, Tan A, et al. The nasal mucosal late allergic reaction to grass pollen involves type 2 inflammation (IL-5 and IL-13), the inflammasome (IL-1β), and complement. Mucosal immunology. 2017;10(2):408-20.

3.	Zhang H, Nestor CE, Zhao S, Lentini A, Bohle B, Benson M, et al. Profiling of human CD4+ T-cell subsets identifies the TH2-specific noncoding RNA GATA3-AS1. Journal of Allergy and Clinical Immunology. 2013;132(4):1005-8.

4.	Abbas AR, Baldwin D, Ma Y, Ouyang W, Gurney A, Martin F, et al. Immune response in silico (IRIS): immune-specific genes identified from a compendium of microarray expression data. Genes & Immunity. 2005;6(4):319-31.

5.	Jevnikar Z, Ostling J, Ax E, Calven J, Thorn K, Israelsson E, et al. Epithelial IL-6 trans-signaling defines a new asthma phenotype with increased airway inflammation. J Allergy Clin Immunol. 2019;143(2):577-90.

6.	Simpson JL, Phipps S, Baines KJ, Oreo KM, Gunawardhana L, Gibson PG. Elevated expression of the NLRP3 inflammasome in neutrophilic asthma. Eur Respir J. 2014;43(4):1067-76.

**Supplementary Table S3. Absolute expression levels of ACE2, TMPRSS2 and Furin genes in different airway compartments**

| Sample type | Gene | Min. | 1st Qu. | Median | Mean | 3rd Qu. | Max. |
| --- | --- | --- | --- | --- | --- | --- | --- |
| Sputum | ACE2 | 12.75 | 20.07 | 23.9 | 25.41 | 29.07 | 73.62 |
| Bronchial brushing | ACE2 | 8.73 | 12.15 | 14.28 | 15.8 | 17.81 | 45.22 |
| Bronchial biopsy | ACE2 | 5.97 | 7.54 | 8.28 | 8.99 | 9.91 | 22.01 |
| Nasal brushing | ACE2 | 26.39 | 51.66 | 64.63 | 74.09 | 89.98 | 191.53 |
| Sputum | TMPRSS2 | 12.13 | 23.85 | 29.78 | 40.10 | 43.59 | 200.17 |
| Bronchial brushing | TMPRSS2 | 15.34 | 48.92 | 59.07 | 59.34 | 69.84 | 181.49 |
| Bronchial biopsy | TMPRSS2 | 9.15 | 22.05 | 28.81 | 31.21 | 37.94 | 77.43 |
| Nasal brushing | TMPRSS2 | 160.96 | 424.13 | 473.83 | 500.22 | 558.62 | 897.79 |
| Sputum | FURIN | 61.85 | 212.37 | 303.62 | 333.07 | 415.93 | 1119.42 |
| Bronchial brushing | FURIN | 27.69 | 41.18 | 49.27 | 57.27 | 63.26 | 209.67 |
| Bronchial biopsy | FURIN | 25.86 | 59.78 | 75.67 | 83.09 | 98.04 | 214.96 |
| Nasal brushing | FURIN | 75.72 | 164.08 | 204.6 | 210.56 | 243.75 | 398.9 |

**List of the U-BIOPRED Consortium project team members** 

Uruj Hoda & Christos Rossios, Airways Disease, National Heart & Lung Institute, Imperial College London, UK & Biomedical Research Unit, Biomedical Research Unit, Royal Brompton & Harefield NHS Trust, London, UK; Elisabeth Bel, Faculty of Medicine, University of Amsterdam, Amsterdam, Netherlands;  Navin Rao, Janssen Research and Development, High Wycombe, Buckinghamshire, United Kingdom; David Myles, Respiratory Therapy Area Unit, GlaxoSmithKline, Stockley Park, UK; Chris Compton, Discovery Medicine, GlaxoSmithKline, Stockley Park, UK; Marleen Van Geest, AstraZeneca R&D Molndal, Sweden; Peter Howarth & Graham Roberts, Faculty of Medicine, Southampton University, Southampton, UK and NIHR Southampton Respiratory Biomedical Research Unit, University Hospital Southampton, Southampton, UK; Diane Lefaudeux, European Institute for Systems Biology and Medicine, CNRS-ENS-UCBL, Université de Lyon, France; Bertrand De Meulder, European Institute for Systems Biology and Medicine, CNRS-ENS-UCBL, Université de Lyon, France; Aruna T Bansal, Acclarogen Ltd, St John's Innovation Centre, Cambridge, CB4 0WS, UK; Richard Knowles, Knowles Consulting, Stevenage Bioscience Catalyst, Gunnels Wood Road, Stevenage SG1 2FX, UK; Damijn Erzen, Boehringer Ingelheim Pharma, Germany; Scott Wagers, BioSci Consulting, BioSci Consulting, Maasmechelen, Belgium; Norbert Krug, Immunology, Allergology and Clinical Inhalation, Fraunhofer Institute for Toxicology and Experimental Medicine, Hannover, Germany; Tim Higenbottam, Corporate Clinical Development, Chiesi Pharmaceutics Ltd, Cheadle, UK. Current address: Allergy Therapeutics, West Sussex, UK; John Matthews, Genentech Inc, 1 DNA Drive, South San Francisco, CA 94080-4990, USA; Veit Erpenbeek, Translational Medicine - Respiratory Profiling, Novartis Institutes for BioMedical Research, Basel, Switzerland; Leon Carayannopoulos, Merck Inc. Kenilworth, New Jersey, USA;  Amanda Roberts, UBIOPRED Patient Input Platform, ELF, Sheffield, UK; David Supple, UBIOPRED Patient Input Platform, ELF, Sheffield, UK; Pim deBoer, UBIOPRED Patient Input Platform, ELF, Sheffield, UK; Massimo Caruso, Department of Clinical and Experimental Medicine Hospital University, University of Catania, Italy; Pascal Chanez, Département des Maladies Respiratoires, Laboratoire d'immunologie, Aix Marseille Université Marseille, France; Sven-Erik Dahlen, The Centre for Allergy Research, The Institute of Environmental Medicine, Karolinska Institute, Stockholm, Sweden; Ildikó Horváth, Department of Pulmonology, Semmelweis University, Budapest, Hungary; Nobert Krug, Fraunhofer Institute for Toxicology and Experimental Medicine Hannover, Germany; Jacek Musial, Dept. of Medicine, Jagiellonian University Medical College, Krakow, Poland; Thomas Sandström, Dept of Medicine, Respiratory and Allergy unit, University Hospital, SE 901 85 Umeå, Sweden.
